# The Food Requirement of Healthy Children

**Published:** 1917-07

**Authors:** 


### THE FOOD REQUIREMENT OF HEALTHY CHILDREN

So much attention has recently been devoted to the problems of nutrition and the energy transformations of the adult body that the requirements as expressed in units of food fuel are beginning to be better understood both by the professions which have to deal with diet and by the laity. It would be an exaggeration to say that many persons are qualified to speak of daily food in terms of calories as they discuss gallons of gasoline or tons of coal in connection with other affairs of daily life. But there are signs of a more tolerant attitude toward the new language in which human dietary needs are best expressed. The exigencies of the present moment have served to focus attention more firmly than ever on what the nutritive needs of living bodies really involve.

For the long period of childhood and adolescence the available information is still far from adequate; and what is known has not yet become as readily accessible as it ought to be. A few years ago it was customary, in calculating dietary needs, to figure the portion belonging to the children of the family in terms of some roughly fixed fraction of the daily allowance for grown-ups. There is, however, an enormous range of variation in the food needs of children occasioned by the unlike physiologic status of these individuals at different ages and stages of growth. Age, size and activity occasion distinctive demands which have come into bolder prominence since the relief of enormous numbers of children of all descriptions has become a national duty in many parts of the world.

It has been said that when food is unrestricted, and the appetite is of the healthy juvenile character, there need be little fear of undernourishment. But delicate children do not always have a normal appetite; and where the food distribution is controlled as it is in public institutions, when a free choice or unlimited supply is not always permitted, the possibility of error expressed in malnutrition or retarded growth is ever present. It is peculiarly timely, therefore, to have a summary of the best evidence now available, such as has just been published by the New York Association for Improving the Condition of the Poor.* Its avowed object is to furnish a survey of the literature, observations, and experiments pertaining to the food requirements of children so that it will become easier to make an intelligent allowance for the adequate nourishment of children and at the same time to reach as many needy families as possible. As the author says, "while it is apparent that insufficient food may result in serious harm to the children, it is equally clear that an unnecessarily liberal allowance to some families may mean the denial of food to many others where thousands of dollars are used in relief."

The statistics collated in the New York survey of the evidence regarding food allowances for healthy children represent the indications furnished by three independent types of investigation, namely: (1) dietary, or observation studies in which the weight of the food eaten by the subject was recorded for a given period of time, and the food values either determined by analysis, or more commonly calculated from figures representing average composition; (2) metabolism or balance experiments in which the intake and output were compared by determining chemically the composition of both the food eaten and the excretory products, thus showing the amount of nitrogen retained in the body for growth of protein tissue, and (3) respiration experiments in which the actual heat produced by the body was either measured directly in an air-tight chamber impervious to heat, or calculated from the amount of oxygen consumed and the carbon dioxid exhaled. Since it is known that the oxidation of food yields energy, it follows that a measurement of the oxidation of foodstuffs in the body will give

* Gillett, Lucy H.: A Survey of Evidence Regarding Food Allowances for Healthy Children, Publication 115, New York Association for Improving the Condition of the Poor, 1917.

the amount of energy produced. This is usually and most conveniently expressed in calories.

In a general way the available data furnished by these three somewhat unlike methods of approach are quite similar. The averages may be summarized as in Table 1:

TABLE 1—CALORIE REQUIREMENT OF CHILDREN AT DIFFERENT AGES

| | Calories | |
|---|---|---|
| Ages, Years | Boys | Girls |
| From 2 to 5 | 1,309 | 1,245 |
| From 6 to 9 | 1,797 | 1,575 |
| From 10 to 13 | 2,337 | 2,015 |
| From 14 to 17 | 2,534 | 2,253 |

It will be noted that there is a difference between the requirements for the children of the two sexes. It might be thought that this is due solely to differences in size at the same age. This seems less likely at present than the assumption of unlike bodily activities, on which a part, at least, of the different needs depends. It is believed that the two sexes have the same basal requirement; that is, the amount of energy required for the maintenance of such internal activities as respiration, heart beat and circulation of the blood, and attainment of suitable muscular tone is comparable.

Age is the largest factor in determining the food requirements of the young. Per unit of weight the amount of energy required for "the mere processes involved in remaining alive"—in other words, the basal need—decreases with increase in age. Although these facts have long been recognized, the unusually conspicuous activities of growing children—a factor added to age and size—have not been so thoroughly appreciated in taking account of the proper food demands of the young. To this must be added further the requisite quota for increment of tissue or growth. Gillett has further pointed out that greatly emaciated children with depleted tissues, which can and ought to be rebuilt rapidly, should receive a more liberal food allowance than would be required by normal children either of the same age or of the same weight. If suitable nutritive conditions are established, recovery or repair can proceed at a speed out of all proportion to the usual rate of growth at the same age.

In view of these considerations, involving features that are highly important rather than novel, we are admonished to adopt the recommendation of Sherman that the food requirement (calories per day) of each member of a family be estimated on his own merits. A small inactive man may require less than a large active boy or girl. This should be recognized and provided for. The upshot of the New York survey has been the compilation of a new set of "Standards" (Table 2) which deserve widespread recognition because of the care and intelligence with which they have been prepared.

TABLE 2—FOOD ALLOWANCES FOR CHILDREN

| | Calories per Day | |
|---|---|---|
| Age, Years | Boys | Girls |
| Under 2 | 900-1,200 | 900-1,200 |
| From 2 to 3 | 1,000-1,300 | 980-1,280 |
| From 3 to 4 | 1,100-1,400 | 1,060-1,360 |
| From 4 to 5 | 1,200-1,500 | 1,140-1,440 |
| From 5 to 6 | 1,300-1,600 | 1,220-1,520 |
| From 6 to 7 | 1,400-1,700 | 1,300-1,600 |
| From 7 to 8 | 1,500-1,800 | 1,380-1,680 |
| From 8 to 9 | 1,600-1,900 | 1,460-1,760 |
| From 9 to 10 | 1,700-2,000 | 1,550-1,850 |
| From 10 to 11 | 1,900-2,200 | 1,650-1,950 |
| From 11 to 12 | 2,100-2,400 | 1,750-2,050 |
| From 12 to 13 | 2,300-2,700 | 1,850-2,150 |
| From 13 to 14 | 2,500-2,900 | 1,950-2,250 |
| From 14 to 15 | 2,600-3,100 | 2,050-2,350 |
| From 15 to 16 | 2,700-3,300 | 2,150-2,450 |
| From 16 to 17 | 2,700-3,400 | 2,250-2,550 |

Quoting the report, the limits given make allowance for individual variation. If a child is tall and growing rapidly at six years of age, he may—and probably will—require 1,600 calories. If of smaller frame, an allowance of only 1,400 or 1,500 calories will be necessary with a normal amount of activity. If he is both large for his age and very active, he will doubtless require the upper limit of the allowance, 1,700 calories. It will be seen that this range of calories may be used to provide for the actual needs of each individual child at any age.—*Journal American Medical Association.*

